# A Single siRNA Suppresses Fatal Encephalitis Induced by Two Different Flaviviruses

**DOI:** 10.1371/journal.pmed.0030096

**Published:** 2006-02-14

**Authors:** Priti Kumar, Sang Kyung Lee, Premlata Shankar, N Manjunath

**Affiliations:** 1The CBR Institute for Biomedical Research and Department of Pediatrics, Harvard Medical School, Boston, Massachusetts, United States of America; **2**Department of Bioengineering, Hanyang University, Seoul, Korea; National Institutes of HealthUnited States of America

## Abstract

**Background:**

Japanese encephalitis virus (JEV) and West Nile virus (WNV) are neurotropic flaviviruses that can cause acute encephalitis with a high fatality rate. Currently there is no effective treatment for these infections.

**Methods and Findings:**

We tested RNA interference (RNAi)-based intervention to suppress lethal JE and WN encephalitis in mice. To induce RNAi, we used either lentivirally expressed short hairpin RNA (shRNA) or synthetic short interfering RNA (siRNA). As target, we selected the cd loop-coding sequence in domain II of the viral Envelope protein, which is highly conserved among all flaviviruses because of its essential role in membrane fusion. Using as a target a species-specific sequence in the cd loop that is conserved only among the different strains of either JEV or WNV, we could achieve specific protection against the corresponding virus. However, by targeting a cross-species conserved sequence within the cd loop, we were able to protect mice against encephalitis induced by both viruses. A single intracranial administration of lentivirally delivered shRNA or lipid-complexed siRNA before viral challenge or siRNA treatment after viral challenge was sufficient for protection against lethal encephalitis.

**Conclusions:**

RNAi-based intervention affords near complete protection from both JEV- and WNV- induced encephalitis in mice. Our results show, to our knowledge for the first time, that siRNA can be used as a broad-spectrum antiviral agent for treating encephalitis caused by multiple related viruses.

## Abstract 2

Intracranial administration to mice of lentivirally delivered short hairpin RNA or lipid-complexed short interfering RNA provides protection against lethal flavivirus encephalitis.

## Introduction

Flaviviruses are small (40–60 nm) enveloped viruses with a single-stranded positive–sense RNA genome that is approximately 11 kb long. The genomic RNA encodes a single polyprotein that is processed into three structural and seven nonstructural proteins [ 1]. The mosquito-borne flaviviruses such as the Japanese encephalitis virus (JEV) and West Nile virus (WNV) are among the most important examples of emerging and resurging pathogens. Japanese encephalitis virus is responsible for ∼50,000 cases of encephalitis world wide annually with 30% mortality and permanent neurological disabilities in 50% of survivors [ 2]. JEV is prevalent in Southeast Asia but has the potential to spread to the New World [ 3]. WNV, once confined to Africa and the Middle East, was introduced into the Americas with 66 cases reported in New York in 1999. Since then, WNV rapidly spread throughout the continental US, and by 2003, 45 states were involved, with 9,858 reported infections and over 2,800 cases of meningitis/encephalitis [ 4, 5]. Currently no effective drugs are available to treat flaviviral infections. Once the virus invades the central nervous system, the course of infection is very rapid, suggesting that success in developing antiviral treatment modalities hinges on the ability to reduce the viral load early in the infection. Moreover, infections by diverse neurotropic flaviviruses are clinically indistinguishable, which makes it important to develop broad-based therapeutic approaches that are effective against multiple viruses within and across the flaviviral species.

RNA interference (RNAi) was originally described as a natural antiviral mechanism in plants. Here, long double–stranded RNA (dsRNA) is processed by the enzyme dicer into small, 21- to 25-nt dsRNA molecules called short interfering RNAs (siRNAs), which mediate the sequence-specific degradation of the target mRNA (reviewed in [ 6– 13]). However, introduction of long dsRNAs in mammalian cells induces an interferon (IFN) response that results in cell death due to global inhibition of protein synthesis. A major advance in the field occurred with the discovery that synthetic short dsRNA resembling the dicer-processed product could mediate specific gene silencing in mammalian cells without evoking the IFN response [ 14]. Since then, RNAi has emerged as a powerful tool for gene silencing with a potential for therapeutic use in viral infections [ 15– 17]. Several studies have demonstrated that the central nervous system is also amenable to RNAi [ 18– 21].

In this study, we explored the feasibility of using RNAi to suppress encephalitis induced by two different flaviviruses. Our results highlight the feasibility of using RNAi for potential therapy in acute neuronal infections.

## Methods

### Cells and Viruses

Baby hamster kidney cell line (BHK21), the mouse neuronal cell line (Neuro 2a), and Vero cell lines were all obtained from ATCC (Manassas, Virginia, United States) and maintained in DMEM with 10% FCS. The Nakayama strain of JEV and B956 strain of WNV were obtained from ATCC, grown, and plaque titrated using BHK21 cells. LD _50_ for both viruses was determined by inoculating serial dilutions of infected mouse brain lysates into groups of mice as described in [ 22].

### Short Interfering RNA Sequences and Generation of Short Hairpin RNA-Expressing Lentiviral Vector

siRNAs were synthesized by Dharmacon (Lafayette, Colorado, United States). The sense strand sequence of the siRNAs designed to target the *envelope* gene were as follows: FvE ^J^, 5′- GGATGTGGACTTTTCGGGA-3′ (JEV nt 1287–1305); FvE ^W^, 5′- GGCTGCGGACTGTTTGGAA-3′ (WNV nt 1287–1305); and FvE ^JW^, 5′- GGGAGCATTGACACATGTGCA-3′ (JEV nt 1307–1328). To generate a lentiviral vector to express shFvE ^J^, two complementary oligonucleotides incorporating the FvE ^J^ sequence were synthesized as a 21-nt inverse repeat separated by a 9-nt loop sequence and inserted between the U6 promoter and the termination sequences in the lentiviral vector Lentilox pLL3.7 as described by Rubinson et al. [ 23]. A control vector targeting the luciferase gene was also similarly generated using a published sequence (nt 155–173) [ 24]. Lentiviral stocks were generated by cotransfection of the lentiviral vector plasmid along with the helper plasmid pHR′8.9ΔVPR (core protein) and either the pVSV-g or pLTR-RVG envelope construct into 293T cells. After 48 h, culture supernatants were filtered through a 0.45 μm membrane filter (Millipore, Billerica, Massachusetts, United States), aliquoted, and stored at −70 °C. Concentrated virus preparations were made by ultrapelleting the supernatants in an SW28 rotor at 25,000 rpm for 1 h. The virus was suspended in PBS for 3–4 h, aliquoted, and stored at −70 °C. Lentiviral stocks were titrated by inoculating serial dilutions on 293T (when pseudotyped with vesicular stomatitis virus glycoprotein) or Neuro 2a cells (when pseudotyped with the rabies virus glycoprotein [RV-G]) and determining green fluorescent protein (GFP) expression by flow cytometry 2 d later and expressed as transduction units (TU)/ml.

### Cell Lines Stably Expressing Short Hairpin RNA

Overnight cultures of Vero or Neuro 2a cells seeded at 1 × 10 ^5^ cells per well in 24-well plates were spin-infected with lentivirus for 2 h at 2,400 rpm (multiplicity of infection [MOI] of 10) in DMEM containing 10% FCS and 8 μg/ml of polybrene. After 2 h further incubation at 37 °C, fresh medium was added to the cells. After ascertaining the transduction efficiency on the basis of GFP expression by flow cytometry (nearly 100% in both cell lines), the cells were maintained in culture for further experiments. Cells were challenged with JEV at different multiplicities of infection. At different times postinfection, the cells were stained with a JEV-specific antibody ( ATCC) followed by a phycoerythrin-conjugated goat anti-mouse polyclonal antibody (DakoCytomation, Glostrup, Denmark) and examined by flow cytometry.

### Northern Blot to Detect Short Hairpin RNA and Viral RNA Degradation

For Northern blot analysis, 5 μg of total cellular RNA extracted from the transduced cells by the RNeasy mini kit (Qiagen, Valencia, California, United States), were run on a 1% denaturing agarose gel, transferred to a positively charged nylon membrane (BrightStar-plus; Ambion, Austin, Texas, United States) and probed using the Northern Max protocol (Ambion). The JEV probe corresponded to the NS4b gene product of JEV, RT-PCR amplified from JEV-infected BHK21 cellular RNA. The DECA template-beta-actin-probe (Ambion) was used for probing the β-actin mRNA that served as the loading control. The probes were labeled with ^32^P-dATP using the DECA prime II random prime labeling kit (Ambion), purified by NucAway spin columns (Ambion). Production of siRNA in lentivirus-transduced cells was analyzed by modified Northern blot designed to capture small RNAs efficiently as described earlier [ 25].

### Short Interfering RNA Transfection

Neuro 2a cells were seeded in six-well plates at 1 × 10 ^5^ per well for 12–16 h before transfection. Lipid-siRNA complexes were prepared by incubating 200 nM of indicated siRNA with Lipofectamine 2000 (Invitrogen, Carlsbad, California, United States), iFect (Neuromics, Bloomington, Minnesota, United States), or JetSI/DOPE (Avanti Polar Lipids, Alabaster, Alabama, United States) formulations in the appropriate volume as recommended by the manufacturer. Lipid-siRNA complexes were added to the wells in a final volume of 1 ml DMEM. After incubation for 6 h, cells were washed, and reincubated in DMEM containing 10% FCS, and infected with either JEV or WNV 24 h post-transfection. The infection levels were monitored after 72 h by flow cytometry using JEV-specific antibody or WNV-envelope specific monoclonal antibody (Chemicon International, Temecula, California, United States).

### RT-PCR and ELISA to Detect IFN-Inducible Genes and Serum IFN Levels

Total RNA was isolated from homogenized mouse brain tissue with TRIzol Reagent (Invitrogen). A total of 5 μg from each sample was reverse transcribed using the ReactionReady first strand cDNA synthesis kit (SuperArray Bioscience, Frederick, Maryland, United States) according to the manufacturer's instructions. Following reverse transcription, the samples were processed for PCR using the MultiGene-12 reverse transcriptase-PCR profiling kit for mouse IFN response genes (SuperArray Bioscience) according to the manufacturer's instructions. The PCR program consisted of an initial incubation at 95 °C for 15 min to denature the samples followed by 30 cycles of 95 °C for 30 s, 55 °C for 30 s, and 72 °C for 45 s. After completion of PCR, 10 μl of each sample was separated by agarose gel electrophoresis, stained, and scanned as a digital image using a CCD camera. The PCR gene products were quantified by NIH Image J (version 1.32j) software. Values obtained for the test samples were normalized with respect to the GAPDH control and divided by the normalized values obtained with the brain sample from untreated mice to determine the fold increases in mRNA levels for each of the genes. IFN levels in serum and brain samples were quantified by using a mouse type I IFN detection ELISA kit from PBL Biomedical (Piscataway, New Jersey, United States), according to the manufacturer's instructions.

### Mouse Infection

BALB/c mice (Jackson Laboratory, Bar Harbor, Maine, United States) aged 4–6 wk were used for all in vivo experiments. All mouse infection experiments were done in a biosafety level 3 animal facility at the CBR Institute for Biomedical Research and had been approved by the institutional review board. For experiments using lentiviruses, mice were inoculated intracranially (IC) with different doses of lentivirus in 5 μl of PBS through the bregma (4 mm deep vertically into the brain using a Hamilton syringe fitted with a 30-gauge needle) at different times before the flaviviral challenge. The mice were subsequently challenged with different doses of JEV or WNV by IC inoculation through the bregma at the same spot as described above. For experiments using siRNA, siRNAs were complexed with iFect (Neuromics) or JetSI/DOPE (Avanti Polar Lipids) according to the manufacturer's instruction. IC injections of siRNA/lipid complexes and flaviviral challenge were done as described earlier.

### Mouse Tissue Preparation

Mice were euthanized and brains removed and used in various experiments. For detection of neuronal cell infection by flow cytometry, freshly isolated brain specimens were used to make a single-cell suspension by gently teasing with the back of a syringe piston. For virus titrations, brain tissues were homogenized in 10% HBSS-BSA followed by repeated passage through a syringe fitted with a 29-gauge needle at least 20 times at 4 °C to release all intracellular virus. Viral titrations were done as described earlier. In some experiments, the same mouse brain homogenates were inoculated on Neuro 2a cells, cultured for 5 d and examined by flow cytometry for viral antigen expression. For histology, the brain samples were fixed in 10% neutral buffered formalin and embedded in paraffin, and 6-μm horizontal sections were stained with hematoxylin and eosin.

## Results

### Suppression of JEV Infection in a Neuronal Cell Line by Lentivirally Expressed Short Hairpin RNA Targeting the Viral *Envelope* Gene

**Figure 1:**
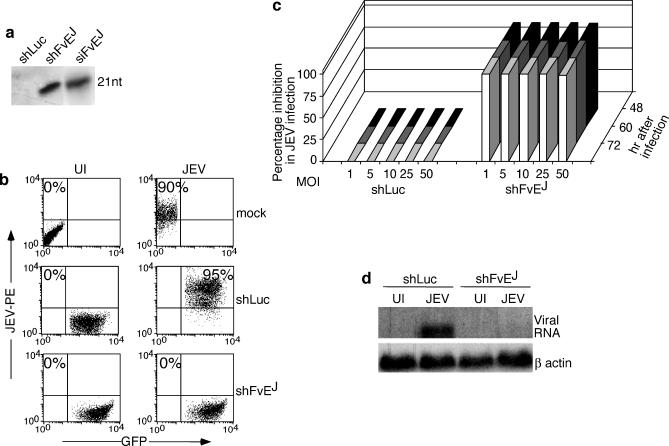
Lentiviral Delivery of FvE ^J^ shRNA Suppresses JEV Replication in Neuro 2a Cell Line (A) Northern blot shows intracellular processing of FvE ^J^ shRNA. RNA extracted from Neuro 2a cells stably transduced with shFvE ^J^ or shLuc lentivirus was probed with ^32^P end-labeled synthetic FvE ^J^ siRNA sense strand to detect intracellular processing of shRNA. Antisense strand of the synthetic FvE ^J^ siRNA (siFvE ^J^) was used as positive control. Before loading, the samples were normalized for total RNA content. (B) shFvE ^J^ inhibits JEV replication in Neuro 2a cells. Mock- or lentivirally transduced Neuro 2a cells were challenged with JEV at a MOI of 1, and the viral replication monitored 60 h later by flow cytometry after staining the cells with a JEV envelope-specific antibody. Percent of infected cells is indicated. The results are representative of at least three independent experiments. (C) Titration of shFvE ^J^-induced inhibition of JEV replication. Neuro 2a cells transduced with shFvE ^J^ or control shLuc lentivirus were challenged with the indicated MOIs of JEV, and viral replication was assessed by flow cytometry at different times postinfection. Percent inhibition of viral replication compared to mock-transduced cells is shown. Results are representative of three experiments. (D) shFvE ^J^ inhibits accumulation of JEV genomic RNA. Total RNA obtained from the control shLuc- or shFvE ^J^ lentivirus-transduced cells, which were either uninfected (UI) or infected with JEV, was probed with JEV- or β-actin cDNA in a Northern blot analysis.

In initial studies, we compared the silencing ability of five siRNAs targeting different regions of the JEV genome and found that a siRNA that targets the *envelope* gene (FvE ^J^, nt 1287–1305 of the genomic RNA) could afford robust protection against JEV infection in cell lines (unpublished data). Moreover, this sequence is completely conserved among all sequenced wild-type JEV isolates. Since the siRNA effect diminishes over time in cell lines because of dilution with cell division, to follow the kinetics of protection we cloned the sequence as a U6 promoter driven template for short hairpin RNA (shRNA) in the lentiviral vector pLL3.7 [ 23]. This vector also contains a GFP gene under the control of the cytomegalovirus promoter, which allows easy monitoring of transduced cells. We used the neurotropic RV-G instead of the conventionally used vesicular stomatitis virus glycoprotein to pseudotype the lentivirus, because it allows retrograde axonal transport to distal neurons and results in more extensive spread of the transduced genes, which would be useful for in vivo applications [ 26]. When the mouse neuronal cell line Neuro 2a was transduced with the RV-G pseudotyped lentivirus encoding FvE ^J^ (shFvE ^J^) or the control luciferase shRNA (shLuc), nearly 100% of the cells were transduced as indicated by GFP expression. However, FvE ^J^-specific short, 21-nt RNA was detected by Northern analysis only in cells stably transduced with shFvE ^J^, but not shLuc ( Figure 1A), indicating the specificity of shRNA expression. To test the ability of the shRNA to inhibit viral replication, the transduced Neuro 2a cells were infected with JEV, and 60 h later the extent of infection was assessed by flow cytometry after staining the cells with a JEV-specific antibody ( ATCC). The high degree of infection seen in the mock- and shLuc-transduced cells was abrogated with shFvE ^J^ transduction ( Figure 1B). We also tested the ability of shFvE ^J^ to protect against challenge with increasing doses of JEV. As Figure 1C shows, shFvE ^J^ exhibited potent antiviral activity in that it abrogated JEV infection in Neuro 2a cells even at a MOI of 50 (highest dose tested). Decreases in the steady-state levels of viral RNA in the shFvE ^J^-transduced cells were also confirmed by Northern analysis using a JEV-specific cDNA probe ( Figure 1D). The antiviral effect of FvE ^J^ shRNA was not due to the induction of an IFN response, because shFvE ^J^ was also able to inhibit viral replication in Vero cells that lack type I IFN genes ( Figure S1A) [ 27]. Moreover, there were no apparent differences in the expression of IFN-responsive genes between shFvE ^J^- and shLuc-transduced cells ( Figure S1B). Thus, shFvE ^J^ effectively inhibits JEV replication by RNAi-mediated degradation of viral RNA.

### Lentivirally Expressed FvE ^J^ shRNA Protects Mice against JEV-Induced Encephalitis

**Figure 2:**
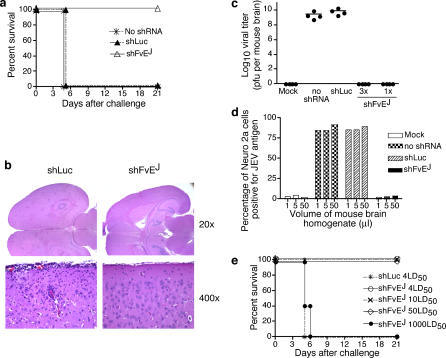
shFvE ^J^ Protects Mice against JEV-Induced Encephalitis (A) BALB/c mice (ten per group) were injected IC on days −4, −2, and 0 with 2 × 10 ^5^ TU of either shFvE ^J^ or shLuc lentiviruses. On day 0, 30 min after injection of the third dose of lentivirus, they were injected at the same spot IC with four LD _50_ of JEV, and the mice were monitored for survival over time. (B) Brain sections from shFvE ^J^ treated mice reveal no flavivirus-induced pathology. Representative photomicrographs of hematoxylin and eosin-stained horizontal brain sections obtained from mice treated with shFvE ^J^ or shLuc lentivirus and infected with JEV for 5 d are shown at indicated magnifications. (C) Lack of infectious virus in the brains of shFvE ^J^-treated mice. Mice were injected with lentiviruses and challenged with JEV as in (A), and their brain homogenates, obtained 5 d after JEV challenge, were plaque-titrated on BHK21 cell monolayers. For shFvE ^J^ lentivirus, viral titers after a single (1×) as well as three (3×) administrations are shown. The viral titers are shown as log plaque-forming units per total brain. Each symbol represents an individual mouse. (D) Brains from shFvE ^J^ treated mice are free of infectious virus. Brain homogenates in (C) were pooled, 1, 10, or 50 μl of pooled homogenate were inoculated onto Neuro 2a cells, and the viral replication was monitored by flow cytometry 5 d later. (E) A single IC injection with shFvE ^J^ is sufficient for protection against JE encephalitis. Mice (five per group) were injected IC with 2 × 10 ^5^ TU of shLuc or shFvE ^J^, challenged 30 min later with indicated doses of JEV, and observed for survival over time.

Next we evaluated the potential of shFvE ^J^ to protect against a lethal IC challenge with JEV. BALB/c mice were injected IC with the control shLuc or shFvE ^J^, pseudotyped with RV-G. All mice were challenged with four lethal doses (LD _50_) of JEV injected at the same site half an hour later and observed for mortality for 21 days. In initial experiments ( Figure 2A) the mice received three IC injections with 2 × 10 ^5^ TU of lentiviruses (the first at 4 d, the second at 2 d, and the third 30 min before JEV challenge). JEV challenge in the control mock- or shLuc-injected mice induced typical symptoms of viral encephalitis including ruffling of fur, hunching, and hind limb weakness beginning on day 4 after infection, which rapidly progressed to paralysis, marked ataxia, and death by the fifth day ( Figure 2A). In contrast, none of the shFvE ^J^-injected mice died or developed any of the clinical symptoms during the entire 21-d period of observation ( Figure 2A). Brain sections from animals challenged with JEV were examined for pathological changes 5 d after infection. Brains of shLuc-treated mice showed the typical histopathological features of a diffuse, disseminated viral encephalitis with hemorrhage, extensive perivascular leukocyte infiltration, and neuronal apoptosis, while no brain inflammation or neuropathology was observed in the shFvE ^J^-treated mice ( Figure 2B). On the fifth day after viral challenge, viral titers in the brain tissue were measured by plaque assays on BHK21 cells. Brain homogenates from the control mice revealed extremely high levels of viral replication, whereas the homogenates from the shFvE ^J^-treated mice remained virus free ( Figure 2C). To further confirm the lack of infectious virus, we inoculated Neuro 2a cell monolayers with increasing amounts of brain homogenates, carried out an extended 5-d culture to allow replication and expansion of virus, and then evaluated flaviviral antigen expression in these cells by flow cytometry. While the Neuro 2a cell cultures inoculated with homogenates from untreated or shLuc-treated mice expressed high levels of viral antigen, viral antigen remained undetectable in cells inoculated with homogenates from shFvE ^J^-treated mice ( Figure 2D). RT-PCR using RNA from the lentivirus-injected brains revealed no apparent differences in the expression of IFN-responsive genes between shFvE ^J^- and shLuc-injected mice ( Figure S1B). Moreover, serum type I IFN protein levels were undetectable by ELISA (unpublished data).

The previous set of experiments showed that three injections of shFvE ^J^ can protect mice against a fatal JEV challenge. We also tested the effectiveness of just one injection of shFvE ^J^ along with viral challenge. While injection with control shLuc did not modify the course of infection, even a single injection of shFvE ^J^ was sufficient to protect mice completely against challenge with four LD _50_ of JEV ( Figure 2E). We also tested the ability of a single injection of shFvE ^J^ to protect against increasing doses of challenge virus. Remarkably, a single injection with shFvE ^J^ afforded complete protection with no detectable virus in the brain homogenates even after challenge with 50 LD _50_ of JEV, although no protection was seen with the unnaturally high dose of 1,000 LD _50_ ( Figure 2E). Collectively these results suggest that shFvE ^J^ can confer a robust RNAi-mediated resistance to fatal Japanese encephalitis. Although the shRNA was coadministered with the challenge virus in these experiments, considering the lag time for the lentivirally transduced vector to be integrated into the host genome and processed into siRNA, the RNAi-mediated antiviral effect is likely to have been activated after JEV replication had already been initiated, suggesting that RNAi may be effective even when administered postinfection.

### FvE ^J^ Synthetic Short Interfering RNA Can Also Protect Mice against Flaviviral Encephalitis

**Figure 3:**
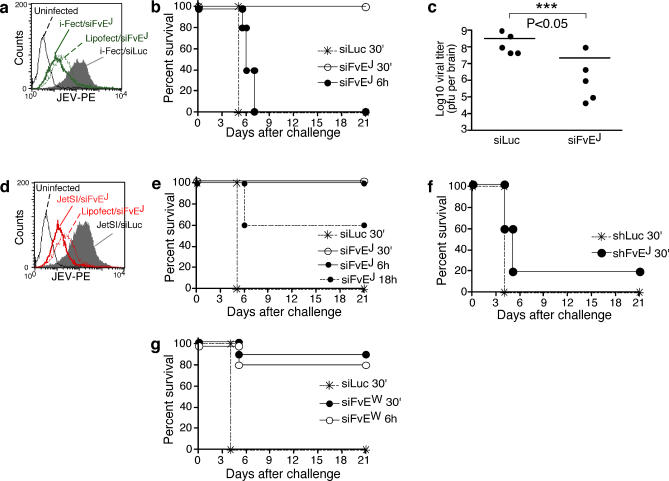
siFvE ^J^ Also Protects against Fatal JEV Infection (A) Transfection of Neuro 2a cells with i-Fect complexed siFvE ^J^ confers protection against JEV infection comparable to lipofectamine transfection. Neuro 2a cells were transfected with siRNA mixed with i-Fect or lipofectamine and after 2 d, they were challenged with JEV at a MOI of ten. Viral replication was monitored 72 h postinfection by flow cytometry. Also shown is an overlay histogram of uninfected Neuro 2a cells and JEV-infected Neuro 2a cells treated prior to infection with either i-Fect/siLuc, lipofectamine/siFvE ^J^, or i-Fect/siFvE ^J^ as indicated. (B) i-Fect-complexed siFvE ^J^ protects mice from JEV infection when injected 30 min but not 6 h after infection. Mice (five per group) were injected IC with four LD _50_ of JEV, and after 30 min or 6 h they were also injected at the same spot with 0.5 nmoles of either siLuc or siFvE ^J^ complexed with i-Fect and monitored for survival over time. (C) i-Fect complexed siFvE ^J^ reduces the level of viral replication in mouse brain when administered 6h post challenge. Mice were injected with siRNAs 6 h after JEV challenge and brain homogenates obtained 3 d later were titrated on BHK21 cell monolayers. Log plaque-forming units per brain is shown. Each symbol represents an individual mouse. (D) Transfection of Neuro 2a cells with JetSI/DOPE complexed siFvE ^J^ results in inhibition of JEV replication. Neuro 2a cells were treated with siFvE ^J^ or siLuc as in a using JetSI/DOPE instead of i-Fect to complex the siRNAs. Overlay histogram denotations are indicated. (E) siFvE ^J^ complexed with JetSI/DOPE protects mice against fatal encephalitis. Mice (ten per group) were injected IC with four LD _50_ of JEV and were treated either with 3.2 nmoles siLuc complexed with JetSI/DOPE after 30 min or with JetSI/DOPE complexed with siFvE ^J^ after 30 min, 6 h, or 18 h after infection and monitored for survival over time. (F) shFvE ^J^ fails to protect against WNV-induced encephalitis. Mice (five per group) were injected with 2 × 10 ^5^ TU of RV-G pseudotyped shLuc or shFvE ^J^ lentiviruses and challenged 30 min later with four LD _50_ of WNV and monitored for survival over time. (G) siFvE ^W^ protects mice against lethal WNV-induced encephalitis. Mice (ten per group) were infected IC with four LD _50_ of WNV, and 30 min or 6 h later they were also injected with 3.2 nmoles of either control siLuc or siFvE ^W^ complexed with JetSI/DOPE, and monitored for survival over time.

Although we observed protection with lentivirally delivered shRNA, this may not be the ideal therapeutic approach, because the long-term effects of lentiviral integration are hard to predict. Moreover, the quantity of siRNA produced endogenously may be limiting for lentiviral delivery to be useful in a clinical setting, as brain cells are likely to contain high levels of replicating virus. On the other hand, similar to drug treatment, synthetic siRNA offers the possibility of escalating the dose for optimal viral suppression and is also potentially safer because of the transient nature of gene silencing. Moreover, siRNA may be particularly well suited for acute infections in which long-term treatment is not needed. However, the poor uptake of duplex siRNA in most mammalian cells in vivo is a major limitation. Although there is a report of the successful use of naked siRNA targeting the pain-related cation channel P2X3 to treat chronic neuropathic pain in a rat model [ 28], other studies suggest that naked siRNA is poorly taken up by brain parenchymal cells [ 29, 30]. Recently, a cationic lipid formulation, i-Fect (Neuromics) has been found to deliver siRNA into neuronal cells without toxicity (Dr. Josephine Lai, University of Arizona Health Sciences Center, personal communication). We tested if synthetic FvE ^J^ siRNA (siFvE ^J^) complexed with i-Fect can protect mice against viral encephalitis. After confirming that i-Fect can transduse siFvE ^J^ siRNA as efficiently as lipofectamine to inhibit JEV infection in Neuro 2a cells ( Figure 3A), we infected mice by IC injection with JEV and, after allowing 30 min for viral adsorption, injected the synthetic siFvE ^J^ or control luciferase siRNA (siLuc) complexed with i-Fect at the same site. All mice injected with siLuc died by day 5, whereas all of the siFvE ^J^-injected mice survived indefinitely ( Figure 3B), suggesting that i-Fect can deliver siRNA into neuronal cells and result in protection that is similar to the lentivirally delivered shRNA.

We also tested if siRNA treatment can protect against an established JEV infection. Mice were first injected with JEV, and siRNA complexed with i-Fect was injected 6 h later, a time point at which the viral RNA is being actively synthesized in the infected cells [ 31]. Under these conditions, although siFvE ^J^ was not able to prevent death, it was able to delay it by 2–3 d ( Figure 3B). Moreover, the viral loads in brain tissue from mice treated 6 h postinfection were two logs lower than in control mice, when tested on day 3 postinfection ( Figure 3C), indicating that siFvE ^J^ complexed with i-Fect could provide partial, but not complete protection when administered postinfection. It should be pointed out that the available i-Fect formulation only allowed us to inject a total of approximately 6 μg (0.5 nmoles) of siRNA in the volume small enough to be safely injected by the IC route. Thus, it is possible that the limited amount of siRNA may not have spread sufficiently to protect cells distant from the site of infection, which is critical for complete protection postinfection. If this were true, injection of a higher dose of siRNA should protect at later time points. To test this, we used another combination cationic lipid formulation, JetSI and the fusogenic lipid dioleoylphosphatidyl-ethanolamine (DOPE), which has also been recently reported to deliver siRNA to brain cells in vivo without toxicity [ 32]. This formulation allowed us to inject higher amounts of siRNA in a small volume. After ascertaining that JetSI/DOPE can successfully deliver siFvE ^J^ into Neuro 2a cells to inhibit JEV replication ( Figure 3D), we injected approximately 40 μg (3.2 nmoles) of siRNAs, complexed with JetSI/DOPE 30 min, 6 h, or 18 h after infection. All control mice injected with siLuc died within 5 d. In contrast, in the siFvE ^J^-treated group, all animals treated with siRNA 30 min or 6 h postinfection, and 60% of mice treated with siRNA 18 h after infection, survived indefinitely ( Figure 3E). As with i-Fect/siFvE ^J^-treated mice, neither IFN-responsive genes nor IFN levels were increased after JetSI/DOPE/siFvE ^J^ treatment compared to JetSI/DOPE/siLuc treatment (unpublished data). Moreover, the surviving siFvE ^J^-treated mice were completely healthy and brain sections taken 21 days after challenge showed no histopathological alterations, suggesting that the treatment was nontoxic (unpublished data).

Next we tested if siRNA targeting the viral *envelope* gene can also suppress WNV. However, upon analysis, the B956 strain of WNV used in the study was found to contain five nucleotide mismatches compared to the FvE ^J^ sequence chosen from JEV. In fact, we found that lentivirally administered shFvE ^J^ offers little protection from WN encephalitis ( Figure 3F). The inability of shFvE ^J^ to protect against a mismatched WN target reinforces our data that the siRNA protects from JEV infection by RNAi rather than by nonspecific induction of IFN. To test if a fully matched siRNA protects against WN, we designed a siRNA targeting the region corresponding to FvE ^J^, but with nucleotides matched completely with the WNV B956 sequence (FvE ^W^). This sequence is also highly conserved in all the sequenced strains of WNV. After confirming that FvE ^W^ siRNA (siFvE ^W^) inhibited WNV replication effectively in vitro (unpublished data), we used the siRNA for in vivo studies. Control siLuc or siFvE ^W^ complexed with JetSI/DOPE was injected at 30 minutes or 6 h after infection with WNV and the mice were observed for mortality. While all the mice injected with siLuc succumbed by d 5, nine of ten mice injected with siFvE ^W^ 30 min after WNV challenge, and four of five mice receiving siFvE ^W^ 6 h after WNV challenge, survived indefinitely ( Figure 3G). These results suggest that, similar to siFvE ^J^ treatment for JEV, siFvE ^W^ can protect against WN encephalitis.

### A Single Short Interfering RNA Targeting a Highly Conserved Region in the Viral *Envelope* Gene Can Protect Mice against Both JEV- and WNV-Induced Encephalitis

**Figure 4:**
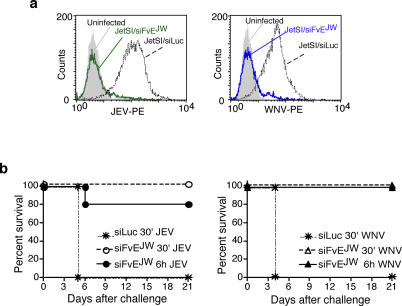
FvE ^JW^ Protects against Encephalitis Induced by Either JEV or WNV (A) FvE ^JW^ siRNA inhibits both JEV and WNV infection in Neuro 2a cells. siRNA-treated Neuro 2a cells were challenged with a MOI of ten of either JEV (left) or WNV (right) and examined for viral replication by flow cytometry 72 h postinfection. Included is an overlay histogram of uninfected Neuro 2a cells and JEV- or WNV-infected Neuro 2a cells transfected prior to infection with either JetSI/DOPE/siLuc or JetSI/DOPE/siFvE ^JW^ as indicated. (B) FvE ^JW^ siRNA protects mice against both JEV and WNV-induced encephalitis. Mice were injected IC with four LD _50_ of JEV (left) or WNV (right), and after 30 min or 6 h they were also injected at the same spot with 3.2 nmoles of either siLuc or FvE ^JW^ complexed with JetSI/DOPE and monitored for survival over time. Ten and five mice per group were used to test the effect of siRNA 30 min and 6 h postinfection, respectively.

We reasoned that it should be possible to design a common siRNA that can suppress both JEV and WNV. The flaviviral envelope glycoprotein is important in host cell receptor binding as well as in the internalization of the viral genome by membrane fusion. Probably because the fusion event is common to all flaviviruses, the cd loop in domain II of the E protein (aa 98–113), which is the region involved in fusion, is highly conserved among all flaviviruses at the amino acid level [ 33]. Although the FvE ^J^ sequence is also derived from within this region (E protein aa 98–103), it is not completely conserved at the nucleotide level and as mentioned earlier, compared to JEV, the WNV strain that we used has multiple nucleotide changes. However, another region in the d loop (E protein, aa 105–111) is highly conserved between JEV, WNV as well as St. Louis encephalitis virus even at the nucleotide level. Thus we designed a 21-nt siRNA (FvE ^JW^), which is identical in sequence between the two viruses except for positions 3 and 21 in JEV and WNV target sequence, respectively. Mismatches at these positions are reported to be well tolerated with no significant effect on siRNA efficacy [ 34]. This siRNA was first tested for its ability to suppress both JEV and WNV in the Neuro 2a cell line. In these cells, siFvE ^JW^ was found to be as effective as siFvE ^J^ or siFvE ^W^, respectively, in suppressing the replication of JEV as well as WNV ( Figure 4A). We also evaluated the ability of siFvE ^JW^ to cross-protect against a lethal challenge with JEV and WNV. Mice were first challenged with either JEV or WNV and, after 30 min or 6 h, injected IC with 3.2 nmoles of siFvE ^JW^ complexed with JetSI/DOPE. All mice injected with the control siLuc, whether challenged with JEV or WNV, died ( Figure 4B). In contrast, all mice injected with siFvE ^JW^ 30 min after infection with either JEV or WNV survived indefinitely. When siFvE ^JW^ was injected 6 h after challenge, 100% of mice challenged with WNV and 80% of mice challenged with JEV survived ( Figure 4B). Collectively these results indicate that the conserved siFvE ^JW^ can confer protection against both JE and WN-induced encephalitis even when administered postinfection.

## Discussion

Here we report the identification of conserved siRNA sequences that can potently protect mice against lethal encephalitis induced by two neurotropic flaviviruses, JEV and WNV. We also demonstrate that siRNA complexed with certain lipids can be used for effective brain delivery. These results highlight the considerable therapeutic potential of RNAi for treating viral encephalitis. Our results also show that by careful design of conserved target sites, it may be possible to use a single siRNA to suppress infection by related viruses across species. This will be particularly important in treating acute and fatal viral infections, where the clinical symptoms often overlap and time does not permit exact etiologic diagnosis.

Although we have demonstrated effectiveness only against JEV and WNV, the siFvE ^JW^ is also likely to be effective in treating St. Louis encephalitis, because the target sequence is very well conserved in all strains of SLE virus. Interestingly, siFvE ^JW^ suppressed infections by both JEV and WNV although there was a single mismatch in the respective target sequence at positions 3 and 21, suggesting that these mismatches are well tolerated. These results are in agreement with several recent studies showing that while there is a stringent homology requirement for the critical central residues, many peripheral nucleotide changes are well tolerated [ 34, 35]. In fact, a detailed study by the Tuschl group has demonstrated that base pairing of the central 13 nucleotides of the siRNA is required for activity, but target mismatches at four nucleotides at either end is tolerated [ 35]. We have not compared RNAi activity with perfectly matched sequences, and thus it is possible that we might be able to further increase the potency using 100% matched siRNAs. However, the siFvE ^JW^ we used was potent enough to suppress viral encephalitis by both viruses. Recently, a non-RNAi-based antisense approach has also been used to inhibit replication of multiple flaviviruses in cell lines [ 36]. In this study, phosphorodiamidate morpholino oligomers were used to target highly conserved sequences in the 5′ and 3′ UTRs of the viral genome. Thus, these regions could also serve as potential therapeutic targets for RNAi-based intervention in flaviviral infections.

An earlier study has also demonstrated partial protection against WNV challenge in a murine model by a siRNA that targeted a different region in the viral *envelope* gene [ 37]. However, unlike our siFvE ^JW^, the sequence of the siRNAs used in that study is not conserved in JEV or St. Louis encephalitis virus, and they are unlikely to protect across species. Moreover, the peripheral route of viral challenge used in that study does not consistently induce encephalitis in mice, which makes it difficult to assess the treatment efficacy accurately. Using the central nervous system route of viral infection that invariably culminates in fatal encephalitis in mice, we have shown that siFvE ^JW^ can afford near complete protection against both JE and WN encephalitis.

Importantly, we were also able to use the siRNAs effectively to suppress WNV postinfection. Intriguingly, based on in vitro studies in cell lines it has been postulated that actively replicating WNV may be resistant to cytoplasmic delivery of siRNA, probably because the viral RNA is sequestered within specialized membranous structures [ 38]. However, in that study, which used an in vitro approach, the failure of the siRNA to protect postinfection was not uniform and was observed only when the cells were transfected with the TKO transfection reagent, and, as the authors themselves pointed out, disparity among results may be based on the type of transfection reagent used. The fact that we consistently observed protection postinfection with both JEV and WNV suggests that the lipid-complexed siRNA is able to penetrate the putative viral RNA encased membranes. Alternatively, the mechanism of viral replication may differ between primary neuronal cells and cell lines.

Our results suggest that a single treatment with siRNA is all that is required for protection against fatal encephalitis, which is encouraging from a therapeutic angle. This may be due to the fact that the siRNA effect is prolonged for up to three weeks in the nondividing neuronal cells [ 39]. Importantly, we noted that a single administration of siRNA could provide ∼60% protection even when administered 18 hours after infection. This is significant because the burst phase for JEV and WNV replication is 18 hours, at which time a large number of progeny virus is released [ 31] that can rapidly spread to distal regions of the brain. It is likely that the siRNA was able to diffuse from the injection site and thus protect the neighboring cells.

Although our results suggest that a single lipid-based siRNA delivery in the brain parenchyma results in some degree of lateral spread and offers protection even in an established infection, this approach is unlikely to work when the infection has spread extensively to involve the entire brain. Ensuring the presence of siRNA throughout the brain will be crucial if the potential of siRNA as a therapeutic drug is to be realized in a clinical setting, where drug administration can begin only after the appearance of clinical symptoms. Thus, it is important to develop improved delivery methods. One approach may be to use continuous intrathecal or intraventricular infusion with lipids and/or targeting with brain receptor-specific antibodies. In fact, these methods have been successfully used in other circumstances [ 32, 40]. Moreover, pegylated immunoliposomes coated with transferrin receptor antibody has been successfully used for brain delivery of shRNA via the intravenous route [ 19, 41]. With any of these methods, even if some degree of reduction in viral load is achieved early in infection, the attenuation would increase the window period available for an immune response to develop that might eventually clear the infection. Although viral mutations even at the conserved sequence is a possibility, given the short time course of viral encephalitis, this is unlikely to be a major limiting factor.

In summary, our study provides optimism for translation of the relatively new RNAi technology from a laboratory tool into a viable clinical strategy for treating acute and deadly viral infections.

## Supporting Information

Figure S1 FvE ^J^ Does Not Induce a Type I IFN Response
(A) Mock- or lentivirally transduced Vero cells were challenged with JEV at a MOI of 10, and viral replication monitored 72 h later by flow cytometry after staining with a JEV-specific antibody. Percentage of infected cells is indicated.
(B) cDNA prepared from Neuro 2a cells stably transduced with shLuc- or shFvE ^J^- (left), or shLuc- or shFvE ^J^- injected mouse brains obtained 4 h (middle) or 24 h (right) after injection were subjected to RT-PCR to measure the induction of IFN-response genes. The PCR products were quantified by NIH Image J (version 1.32j) software. Normalized values obtained for the test samples were divided by that obtained with untreated Neuro 2a cells or untreated mouse brains to determine the changes in mRNA levels for each of the genes.
(33 KB PDF).

### Accession numbers

The sequences of the viral strains used in this study are listed in GenBank ( http://www.ncbi.nlm.nih.gov/entrez/) and their accession numbers are U03694 (JEV, Nakayama strain) and AY532665 (WNV, B956 strain).

**Patient Summary**

**Background**

There are number of viruses that can cause encephalitis (inflammation of the brain). Two such viruses are West Nile virus and Japanese encephalitis virus, part of a family of viruses called flaviviruses, which are transmitted by mosquitoes and ticks. Other diseases caused by flaviviruses are yellow fever (for which the viruses are named—“flavus” is Latin for yellow), and dengue. Japanese encephalitis virus occurs in Southeast Asia, causing 50,000 cases each year. West Nile virus originated in Africa and the Middle East, but is now in the Americas. Encephalitis caused by these viruses is often very severe and can be fatal. There are no specific treatments for the encephalitis caused by these viruses.

**Why Was This Study Done?**

Recently researchers have started to use a technique known as RNA interference (RNAi) to silence the expression of specific genes. RNAi was originally described as a natural antiviral mechanism in plants. RNA is the normal intermediary between the DNA and proteins. Small stretches of complementary RNA, called short interfering RNA (siRNA) can be made synthetically and introduced into cells to specifically target RNA and thus silence particular genes.

**What Did the Researchers Do and Find?**

The researchers identified areas of similar sequence within genomes of two flaviviruses. They manufactured short interfering RNA specific for each of these viruses, and also another common siRNA that could target both viruses equally well. When the siRNA was injected into the brain before, at the same time as, or after infection with the virus, it protected mice against infection with the appropriate virus. In addition, the common RNA could protect mice against encephalitis induced by both viruses.

**What Do These Findings Mean?**

This study in mice shows that in principle it might be possible to use the technique of RNAi to protect against encephalitis caused by these viruses. However, in this study the treatment was administered only at early time points after infection. Thus, further studies will need to be done to see if it can work when given much later in the course of the disease. In addition, before it can be used in human disease it will be necessary to develop ways to give the interfering RNA to humans, and to test the safety and effectiveness of the approach.

**Where Can I Get More Information Online?**

MedlinePlus has a page with links to information on encephalitis:

http://www.nlm.nih.gov/medlineplus/encephalitis.html

and West Nile virus:

http://www.nlm.nih.gov/medlineplus/westnilevirus.html

Wikipedia, the free Internet encyclopedia that anyone can edit, has a page discussing RNAi with links to other resources:

http://en.wikipedia.org/wiki/RNAi

## Abbreviations

dsRNA: double-stranded RNA
E: envelope
GFP: green fluorescent protein
IC: intracranial challenge
IFN: interferon
JE(V): Japanese encephalitis (virus)
MOI: multiplicity of infection
RNAi: RNA interference
RV-G: rabies virus glycoprotein
shRNA: short hairpin RNA
siRNA: short interfering RNA
TU: transduction units
WN(V): West Nile (virus)
