# Supplementary material for: A Single siRNA Suppresses Fatal Encephalitis Induced by Two Different Flaviviruses
Source: PLoS Med. 2006 Feb 14;3(4):e96. doi: 10.1371/journal.pmed.0030096 (PMC1361782; doi:10.1371/journal.pmed.0030096)
Supplement: Supplementary file 1 [file pmed.0030096.sg001.pdf]

FIG S1

a

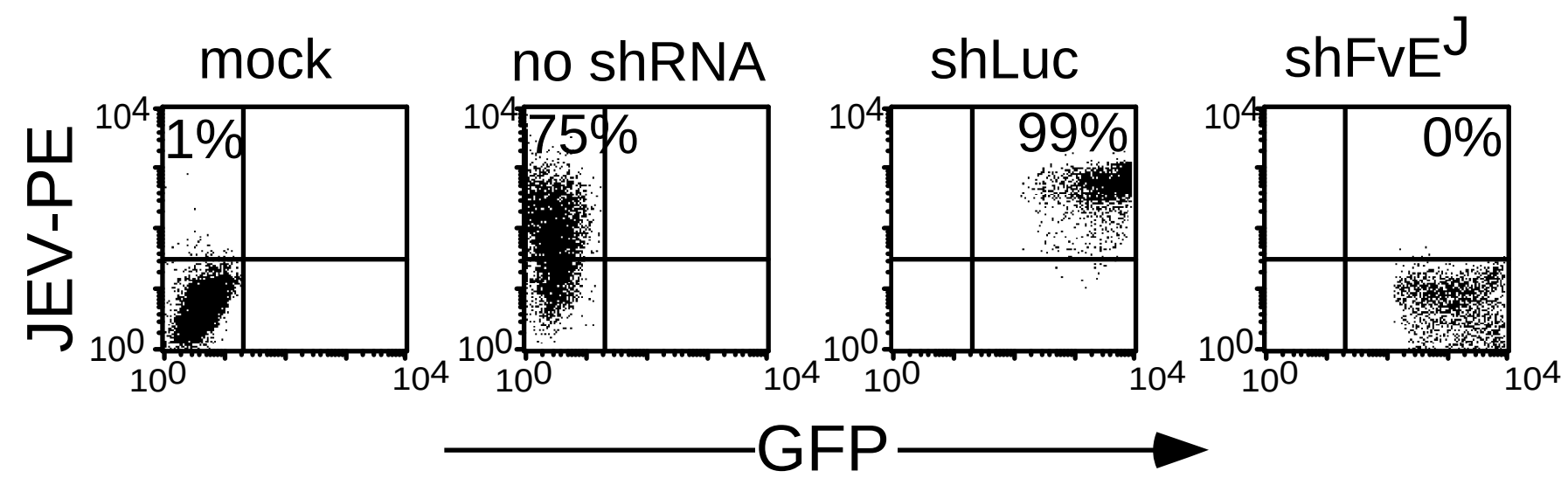
mock
no shRNA
shLuc
shFvEJ
JEV-PE
1%
75%
99%
0%
10^4
10^0
GFP

b

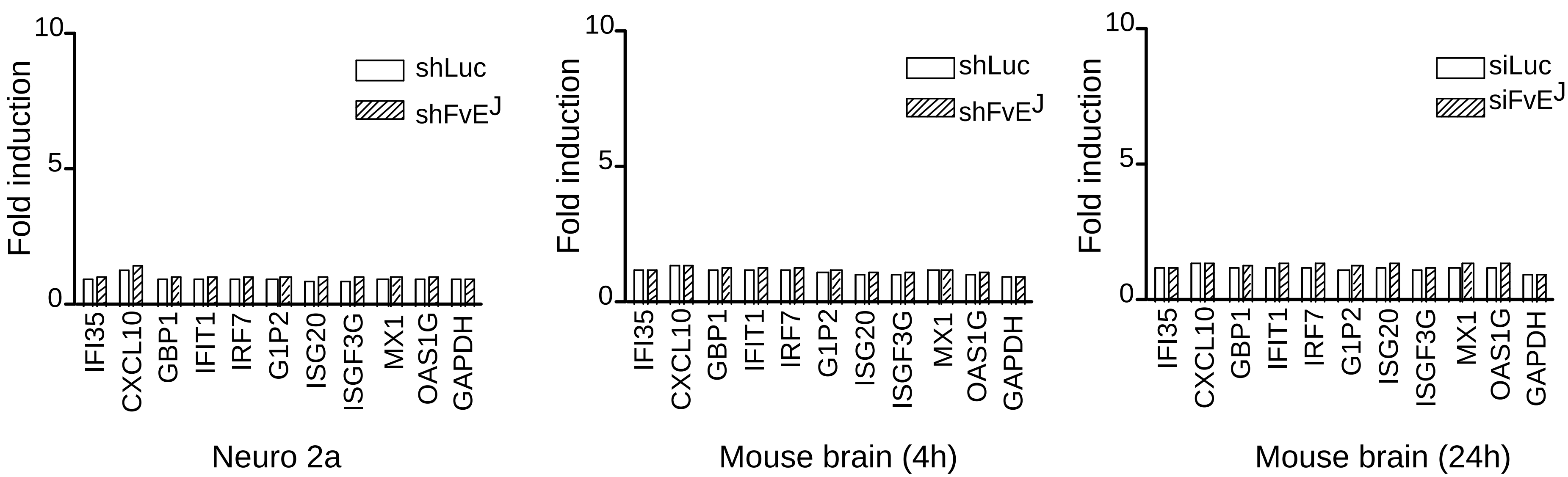
Fold induction
10
5
0
shLuc
shFvEJ
siLuc
siFvEJ
IFI35
CXCL10
GBP1
IFIT1
IRF7
G1P2
ISG20
ISGF3G
MX1
OAS1G
GAPDH
Neuro 2a
Mouse brain (4h)
Mouse brain (24h)
